# Molecular Interactions and Antioxidant Properties of White Wine Phytochemicals: A Mechanistic Study of Serum Protein Binding

**DOI:** 10.3390/biom16081153

**Published:** 2026-08-07

**Authors:** Dinorah Barasch, Alina Nemirovski, Emmanuelle Merquiol, Joseph Deutsch, Dejian Huang, Pitipong Thobunluepop, Alma Leticia Martinez-Ayala, Patricia Arancibia-Avila, Fernando Toledo-Montiel, Paweł Paśko, Shela Gorinstein

**Affiliations:** 1 Institute for Drug Research, School of Pharmacy, Faculty of Medicine, The Hebrew University of Jerusalem, Jerusalem 9112001, Israel; dinorah.barasch@mail.huji.ac.il (D.B.); alina.nemirovskai@mail.huji.ac.il (A.N.); emmanuellem@savion.huji.ac.il (E.M.); 2 Department of Food Science & Technology, National University of Singapore, Singapore 117542, Singapore; dejian@nus.edu.sg; 3 Department of Agronomy, Faculty of Agriculture, Kasetsart University, Chatuchak, Bangkok 10900, Thailand; fagrppt@ku.ac.th; 4 Centro de Desarrollo de Productos Bióticos, Instituto Politécnico Nacional, Yautepec 62731, Morelos, Mexico; alayala@ipn.mx; 5 Departamento de Ciencias Básicas, Facultad de Ciencias, Universidad del Bío-Bío, Chillán 378 000, Chile; parancib@ubiobio.cl (P.A.-A.); ftoledo@ubiobio.cl (F.T.-M.); 6 Department of Food Chemistry and Nutrition, Faculty of Pharmacy, Jagiellonian University Medical College, 30-688 Kraków, Poland; p.pasko@uj.edu.pl

**Keywords:** human serum albumin, white wine phenolics, molecular docking, polyphenol-protein interaction, fluorescence quenching

## Abstract

This study investigated the interactions between phenolic compounds from Israeli and Chilean white wines and human serum carrier proteins, including human serum albumin (HALB), gamma-globulin (HGLO), and fibrinogen (HFB), to characterize their antioxidant capacity and serum protein-binding behavior under controlled experimental conditions. The analyzed wines included Israeli Chardonnay (ICR), Chilean Chardonnay (CCR), Israeli Sauvignon Blanc (ISB), and Chilean Sauvignon Blanc (CSB). HPLC and FTIR fingerprinting revealed cultivar- and region-dependent differences in phenolic composition, with Chardonnay wines showing stronger protein-binding behavior and Sauvignon Blanc samples displaying high antioxidant efficiency relative to their phenolic content. ICR exhibited the highest total binding capacity, 46.44%, and the strongest albumin interaction, with a binding constant (Kb) of 8.44 × 10^4^ M^−1^ and a Gibbs free energy (ΔG) value of −35.03 kJ/mol. Empirical fluorescence quenching kinetics demonstrated that white wine phenolics establish stable physical complexes with human serum proteins, displaying a distinct preferential affinity for HALB as the protein showing the strongest apparent interaction among the proteins tested. Two- and three-dimensional fluorescence spectroscopy confirmed substantial quenching of the intrinsic tryptophan and tyrosine residues, indicating meaningful microenvironmental alterations within the protein’s active transport sites. These empirical interactions were closely mirrored by complementary molecular docking simulations, which provided a structural visualization of the physical binding interactions. ICR also showed the highest antioxidant capacity, with DPPH and CUPRAC values of 1.66 and 2.91 mmol TE/L, respectively. Ethanol control showed negligible effects, indicating that the observed bioactivity was mainly associated with the polyphenolic matrix. Among the investigated samples, Chardonnay showed higher apparent protein-binding capacity, whereas Sauvignon Blanc showed relatively high antioxidant efficiency in relation to its phenolic content.

## 1. Introduction

Epidemiological studies have reported an inverse association between moderate wine consumption and the prevalence of coronary artery disease (CAD), a phenomenon traditionally attributed to the high phenolic content of red wine varieties. Although the cardioprotective effects of red wine, including inhibition of platelet aggregation and reduction in low-density lipoprotein (LDL) oxidation, are well documented, the biochemical potential of white wines remains comparatively undervalued [1,2]. Recent evidence, however, suggests that the unique bioactive profile of white wines, characterized by non-flavonoid phenolics such as tyrosol and hydroxycinnamic acids, may play an important role in modulating vascular tone and enhancing flow-mediated dilation, hereby contributing to mechanisms potentially relevant to cardiovascular protection independently of alcohol content [3].

The biological disposition of dietary polyphenols may be influenced by their interactions with major serum proteins, particularly human serum albumin (HALB), globulin (HGLO), and fibrinogen (HFB). These proteins serve as major circulatory transporters, and their interactions with dietary bioactive compounds may influence ligand distribution and availability. However, protein-binding measurements performed under in vitro conditions do not directly establish the metabolic stability, systemic distribution, or biological efficacy of these compounds in vivo. Binding to human serum albumin may influence the apparent solubility, transport, distribution, and availability of dietary polyphenols in circulation. Albumin binding may also limit the immediately available unbound fraction while serving as a reversible circulating reservoir for these compounds. Because many drugs are transported by the same albumin-binding regions, competition between dietary phenolics and albumin-bound drugs represents a theoretically relevant pharmacokinetic mechanism that requires compound-specific experimental evaluation [4,5,6]. In white wines, the structural diversity of the phenolic profile, particularly within the fingerprint region of Fourier-transform infrared spectroscopy (FTIR; 1800–900 cm^−1^), reveals specific C-O and C-C stretching vibrations that may distinguish regional varietals. Despite their lower total polyphenol content compared with red wines, selected hydroxycinnamic acids present in white wines may exhibit favorable binding dynamics at Sudlow’s site I, located in subdomain IIA of HALB, suggesting a potentially relevant albumin-binding mechanism that requires validation under physiologically representative conditions [7,8]. By identifying these functional groups through FTIR fingerprinting, it is possible to link the biological relevance of specific phenolic monomers, such as catechin and gallic acid, with the overall behavior of wine extracts.

Building on our recent research on red and fruit wines [9,10,11], the present study focuses on the still relatively unexplored biochemical properties of Sauvignon Blanc and Chardonnay wines from Israel and Chile.

While evaluating the interaction profiles of white wine extracts across multiple serum proteins provides an initial baseline, a deeper empirical investigation is necessary to understand precise transport dynamics. Consequently, this study utilizes detailed three-dimensional (3D) Excitation–Emission Matrix (EEM) fluorescence spectroscopy focused specifically on human serum albumin to determine quenching mechanisms, utilizing computational docking as a complementary tool to visualize the underlying binding activity. By comparing phytochemical-rich wine extracts with an ethanol control, this study isolates and evaluates the specific contribution of the polyphenolic matrix to targeted protein–polyphenol interactions. This investigation provides a mechanistic framework for comparing the antioxidant capacity and serum protein-binding behavior of phenolic compounds from Israeli and Chilean white wines under controlled experimental conditions. The possible relevance of these interactions to systemic transport or cardiovascular-related processes remains hypothetical and requires confirmation in biological models [12,13].

## 2. Materials and Methods

### 2.1. Reagents and Chemicals

Sigma-Aldrich (St. Louis, MO, USA) supplied most of the high-purity reagents and standards used in this study, including phenolic compounds and components of the antioxidant assays. The phenolic standards included caftaric, caffeic, gallic, tannic, *p*-coumaric, protocatechuic, and vanillic acids, as well as hydroxytyrosol, resveratrol, catechin, and epicatechin. Reagents used for colorimetric and antioxidant analyses included Folin–Ciocalteu reagent, DPPH (2,2-diphenyl-1-picrylhydrazyl), neocuproine (2,9-dimethyl-1,10-phenanthroline), Trolox, iron (III) chloride hexahydrate, and copper (II) chloride dihydrate. The biological proteins human serum albumin (HALB), human gamma-globulin (HGLO), and human fibrinogen (HFB) were also purchased from Sigma-Aldrich (St. Louis, MO, USA). Basic laboratory chemicals, including glacial acetic acid, sodium nitrite, sodium hydroxide, and 37% hydrochloric acid, were of analytical grade. All phenolic stock solutions were prepared in methanol at a concentration of 1 mg/mL and stored at −80 °C to ensure stability.

### 2.2. Material

The sample selection was purposive and designed as a controlled comparative case study rather than as a comprehensive survey of white wines from Israel and Chile. Two widely available cultivars, Chardonnay and Sauvignon Blanc, were selected because they represent contrasting white wine phenolic profiles and were commercially available from producers in both investigated countries. This paired design allowed cultivar- and origin-related differences to be explored while limiting variation associated with vintage and product category. All wines were from the same 2023 vintage and represented comparable commercial products. For each wine, five bottles were purchased from different retail locations. Bottles of the same label, vintage, and production batch, with comparable storage histories and shelf lives, were selected to reduce variation unrelated to cultivar or geographical origin. Each bottle was treated as an independent commercial sampling unit and analyzed separately. This replication was intended to assess the consistency of the selected products and to reduce the influence of an individual bottle; it does not imply representation of the full diversity of either cultivar or country of origin. The Israeli samples, produced by Golan Heights Winery, included Yarden Sauvignon Blanc, 12.5% alcohol by volume (ABV), denoted as ISB, and Yarden Chardonnay, 14.0% ABV, denoted as ICR. The Chilean samples, produced by Santa Carolina Winery, included Santa Carolina Reserva Sauvignon Blanc, 13.0% ABV, denoted as CSB, and Santa Carolina Reserva Chardonnay, 13.8% ABV, denoted as CCR. Upon collection, the wines were transferred into 50 mL sterile conical tubes. Analytical aliquots were refrigerated at 8 °C for immediate testing, whereas backup samples were stored at −80 °C to preserve long-term bioactivity and antioxidant stability. Before experimental analysis, the samples were equilibrated to room temperature and diluted according to the specific requirements of each assay.

### 2.3. Determination of Phenolic Compounds, Total Phenolic Content, Tannins, and Antioxidant Capacity

#### 2.3.1. HPLC Analysis of Specific Phenolics

Quantitative analysis of individual phenolic compounds—including caftaric, caffeic, gallic, tannic, *p*-coumaric, protocatechuic, and vanillic acids, alongside hydroxytyrosol, resveratrol, catechin, and epicatechin—was performed using high-performance liquid chromatography (HPLC) according to previously established protocols [14,15,16]. Briefly, a 50 mL aliquot of each wine sample was subjected to sequential liquid–liquid extraction (LLE), beginning with a triple extraction using 25 mL of diethyl ether to selectively capture less polar aglycones and phenolic acids. This was immediately followed by a triple extraction using 25 mL of ethyl acetate to maximize the recovery of polar monomeric flavonoids and esterified hydroxycinnamates.

Compared to direct aqueous phase analysis, this dual organic solvent extraction approach functions as an essential sample clean-up step, effectively isolating the bioactive phytochemical fraction from highly polar wine matrix components (such as residual reducing sugars and organic acids) that could otherwise foul the stationary phase or cause baseline co-elution. The combined organic extracts were subsequently dried over anhydrous Na_2_SO_4_ for 30 min, filtered through Whatman No. 40 filter paper, and evaporated to dryness under a vacuum. The dry residue was reconstituted in 2 mL of a 1:1 (*v*/*v*) methanol/water mobile-phase-compatible mixture, ensuring complete dissolution of both flavonoid and non-flavonoid fractions for reproducible, high-resolution quantitative injection. Separation was performed using a Waters HPLC system (Waters Corp., Milford, MA, USA) equipped with a 600-MS controller, 717 Plus autosampler, and 996 photodiode-array detector. A Nova-Pak C18 column (30 cm × 3.9 mm i.d.; Waters Corp., Milford, MA, USA) was used with a mobile phase gradient consisting of solvent A (water/acetic acid, 98:2, *v*/*v*) and solvent B (water/acetonitrile/acetic acid, 78:20:2, *v*/*v*/*v*). The flow rate was maintained between 1.1 and 1.2 mL/min over a 120 min run at 20 °C, with an injection volume of 50 µL for all samples. All analyses were performed in five independent replicates.

#### 2.3.2. Total Phenolic Content

Total phenolic content (TPC) was determined using the Folin–Ciocalteu colorimetric method [17]. Briefly, 250 µL of wine sample was mixed with 1250 µL of Folin–Ciocalteu reagent, 10%, *v*/*v*, in water, and 1000 µL of 7.5% sodium carbonate. After incubation for 15 min in a water bath at 50 °C in the dark, absorbance was measured at 765 nm using a Hewlett-Packard 8452A spectrophotometer (Hewlett-Packard Co., Rockville, MD, USA). Results are expressed as milligrams of gallic acid equivalents per liter, mg GAE/L. All analyses were performed in three independent replicates.

#### 2.3.3. Total Tannins

The concentration of total tannins (TNs) was estimated using the vanillin–HCl assay [18]. A 0.5 mL aliquot of wine was reacted with 3 mL of 4% methanolic vanillin and 1.5 mL of concentrated hydrochloric acid. After standing for 15 min, absorbance was measured at 500 nm against a water blank. All analyses were performed in five independent replicates.

#### 2.3.4. Antioxidant Capacity Assays

Two complementary methods were used to evaluate antioxidant capacity, and the results are expressed as millimoles of Trolox equivalents per liter, mmol TE/L [19,20]. For the DPPH assay, 25 µL of sample was mixed with 180 µL of 6 mM DPPH radical solution. The decrease in absorbance at 517 nm was monitored every 30 s over a 10 min period. For the CUPRAC assay, cupric-reducing antioxidant capacity was determined according to established protocols [21,22]. Wine samples were first diluted 1:10, *v*/*v*, with distilled water. Then, 0.5 mL of the diluted wine was mixed with 1.0 mL each of 0.010 M Cu (II), ammonium acetate buffer, pH 7.0, and 0.0075 M neocuproine in ethanol, supplemented with 0.6 mL of distilled water. After incubation for 1 h in the dark, absorbance was measured at 450 nm. All analyses were performed in five independent replicates.

In addition to the directly measured DPPH and CUPRAC antioxidant capacities, two exploratory efficiency indices were calculated to compare antioxidant capacity relative to total phenolic content: antioxidant efficiency (AE) and molar efficiency (ME). A third exploratory index, the Binding-to-Antioxidant Index (BAI), was calculated to relate albumin-binding strength to antioxidant efficiency. These indices were used exclusively for comparative purposes within the investigated wine samples. Complete equations, scaling factors, units, and interpretation criteria are provided in Supplementary Materials.

### 2.4. Fourier-Transform Infrared Spectroscopy of Wine Polyphenols

Fourier-transform infrared spectroscopy (FTIR) was used to characterize the phenolic components present in the white wine extracts. Spectra were recorded using a Nicolet iS10 FTIR spectrometer (Thermo Scientific Instruments LLC, Madison, WI, USA) equipped with a Smart iTR attenuated total reflectance (ATR) accessory. For sample preparation, the isolated white wine extracts were freeze-dried to form a solid powder matrix, and approximately 2 mg of each dried extract was thoroughly mixed with 200 mg of potassium bromide to prepare translucent pellets for mid-infrared analysis. Data were collected over the spectral range of 4000–500 cm^−1^ at a resolution of 4 cm^−1^. Each final spectrum represented the average of 32 individual scans. The results are presented as percentage transmittance versus wavenumber [23].

### 2.5. Fluorescence Quenching, Three-Dimensional Fluorescence Analysis, Protein Binding, Stern–Volmer Kinetics, and Thermodynamic Parameters

The phenolic profiles and protein-binding properties of the wine samples were investigated using two-dimensional fluorescence spectroscopy (2D-FL) with a Jasco FP-6500 spectrofluorometer (JASCO Corp., Tokyo, Japan). For fluorescence profiling, emission spectra were recorded in the range of 310–500 nm at a fixed excitation wavelength of 295 nm [9,10]. The interactions between wine polyphenols and human serum proteins were evaluated using fluorescence spectroscopy. Human serum albumin (HALB) was prepared at a concentration of 1.0 × 10^−5^ mol/L in 0.05 mol/L Tris–HCl buffer, pH 7.4, containing 0.1 mol/L NaCl. Human gamma-globulin (HGLO) and human fibrinogen (HFB) stock solutions were prepared at a concentration of 20 µM in 10 mM phosphate buffer, pH 7.4. All protein solutions were stored at 4 °C before use.

To assess protein-binding activity, the initial fluorescence intensities of the pure protein solutions were recorded as baseline values. These values were then compared with the fluorescence intensities measured after the addition of wine samples. For fluorescence quenching measurements, the interaction between white wine phytochemicals and HALB, HGLO, HFB was monitored at room temperature. The protein concentration was kept constant, whereas wine samples, acting as quenchers, were added at increasing concentrations. Fluorescence spectra were recorded at an excitation wavelength of 280 nm, and emission was monitored between 300 and 450 nm. The resulting decrease in protein fluorescence intensity was used to estimate the binding affinity of wine polyphenols [24].

To further evaluate structural modifications of the primary carrier protein, three-dimensional fluorescence spectra were recorded for HALB in its native state and after the addition of the isolated wine polyphenol extracts using the same Jasco FP-6500 spectrofluorometer. Excitation wavelengths were scanned from 200 to 320 nm at 5 nm intervals, while emission spectra were collected over the range of 200–450 nm. The resulting excitation–emission matrices were used to assess changes in the characteristic fluorescence regions associated with the polypeptide backbone and with tyrosine and tryptophan residues. These data provided complementary information on alterations in the protein microenvironment induced by polyphenol binding. The derived microenvironmental parameters were subsequently integrated into comprehensive multi-parameter radar profiles.

The selectivity index was used to compare the relative affinity of wine polyphenols toward albumin and globulin and determine whether the phenolic compounds preferentially interacted with HALB rather than HGLO.

Fluorescence quenching of HALB with wine phenolics was used for Stern–Volmer approach. The corresponding quenching constant was used as an indicator of quenching efficiency and of the accessibility of wine phenolics to the fluorescent amino acid residues of the protein. The fluorescence results were interpreted together with the molecular docking data, particularly with regard to the possible involvement of Sudlow site I of human serum albumin.

The binding constant and the apparent number of binding sites were determined from the fluorescence quenching data using a double-logarithmic model. The binding constant was interpreted as a measure of the stability of the protein–ligand complex, whereas the number of binding sites reflected the apparent stoichiometry of the interaction [25].

The Gibbs free energy change was calculated to assess the spontaneity of the binding process. Negative values were interpreted as evidence of spontaneous interaction between wine phenolics and the analyzed proteins [26,27].

The complete equations, calculation procedures, definitions of all variables, and details of data interpretation are provided in Supplementary Materials.

### 2.6. Molecular Docking of White Wine Phenolics with Serum Proteins

To elucidate the molecular-level interactions between the identified white wine phenolics and human serum proteins, computational docking analysis was performed using AutoDock Vina (version 1.2.0; The Scripps Research Institute, La Jolla, CA, USA). The study focused on human serum albumin (HALB; PDB ID: 1H9Z) served as the primary target. Albumin was selected as the primary target because it is the major plasma ligand-binding protein and provides a biologically relevant model for investigating protein–phenolic interactions.

High-resolution crystal structures of the target proteins were obtained from the RCSB Protein Data Bank. Protein structures were prepared by removing crystallographic water molecules and heteroatoms. Kollman and Gasteiger charges were assigned, and hydrogen atoms were added using AutoDock MGL Tools (version 1.5.7; The Scripps Research Institute, La Jolla, CA, USA). Hydrogen-bonding networks were optimized before docking. The three-dimensional structures of the major wine phenolics were energy-minimized prior to docking analysis [8].

Molecular docking simulations were used to predict the binding orientation of wine phenolics within the Sudlow site I binding pocket of HALB, located in subdomain IIA. Interaction stability was evaluated based on docking scores, binding free energy values, and the presence of hydrogen bonds and hydrophobic interactions, particularly with key amino acid residues such as Lys-199 and Arg-222.

Specific binding domains were defined to ensure precise ligand placement. For HALB, the grid box was centered on Sudlow site I with dimensions of 30 Å × 30 Å × 30 Å. Docking results were evaluated based on the predicted binding free energy values (ΔG), expressed in kJ/mol, representing the most stable protein–ligand conformations. Binding affinities and the amino acid residues involved in hydrogen bonding and hydrophobic contacts were analyzed using BIOVIA Discovery Studio 2017 R2 Client (Dassault Systèmes BIOVIA Corp., San Diego, CA, USA).

Secondary structure composition, including α-helix and β-sheet content, was predicted using the DSSP (Define Secondary Structure of Proteins) algorithm. This method assigns structural motifs based on hydrogen-bonding patterns and dihedral angles identified in PDB coordinate files of the docked complexes. The analysis enabled quantification of docking-associated structural shifts, such as changes in α-helix content [28,29].

### 2.7. Data Analysis

Statistical analysis of all experimental data was performed using one-way analysis of variance (ANOVA) to determine significant differences among the wine samples. To ensure robust results, five independent bottles of each wine were analyzed, and each sample was measured in five replicates, yielding a total of 25 measurements per parameter.

When significant differences were detected, Duncan’s multiple range test was applied to compare mean values at a 95% confidence level. A *p*-value of ≤0.05 was considered statistically significant. This statistical approach was used to evaluate differences in the phenolic composition, antioxidant capacity, and ligand-binding properties of Israeli and Chilean white wine varieties.

## 3. Results and Discussion

### 3.1. Bioactive Profiles and Antioxidant Efficiency of White Wines

**Figure 1:**
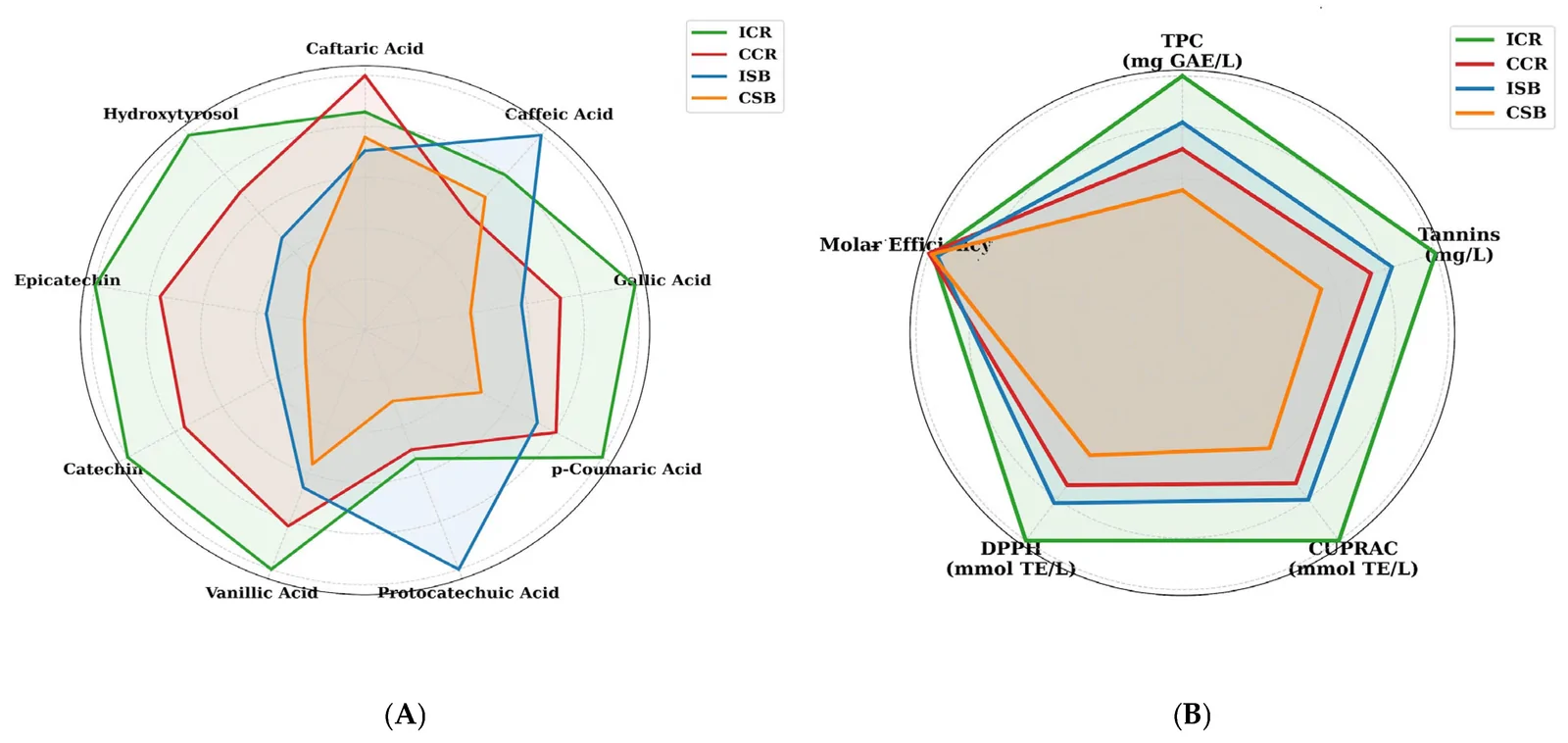
Phytochemical and antioxidant profiles of Israeli and Chilean white wines. (**A**) Radar plot showing the relative distribution of selected phenolic compounds, including caftaric, caffeic, gallic, *p*-coumaric, protocatechuic, and vanillic acids, as well as catechin, epicatechin, and hydroxytyrosol, in the analyzed wine samples. (**B**) Radar plot presenting the comparative bioactive profiles of the wines based on total phenolic content, tannin content, CUPRAC and DPPH antioxidant capacity, and molar efficiency. The corresponding numerical values used to construct panels (**A**,**B**) are provided in Supplementary Tables S1 and S2, respectively. ICR, Israeli Chardonnay; CCR, Chilean Chardonnay; ISB, Israeli Sauvignon Blanc; CSB, Chilean Sauvignon Blanc.

Detailed HPLC profiling (Figure 1A) showed that ICR was particularly enriched in gallic acid, 7.21 mg/L, and catechin, 19.80 mg/L, compared with the other samples. These phenolic compounds may play an important role in protein–ligand interactions due to their hydroxyl groups and aromatic structures. Interestingly, CCR showed the highest concentration of caftaric acid, 42.82 mg/L. Statistical differentiation across most phenolic acids and flavonoids suggests that both regional origin, Israel versus Chile, and cultivar, Chardonnay versus Sauvignon Blanc, contribute to the formation of a distinct phytochemical fingerprint for each wine.

The bioactive profiles of Israeli and Chilean Chardonnay (ICR and CCR) and Sauvignon Blanc (ISB and CSB) wines were characterized also based on total phenolic content (TPC), total tannins, and antioxidant capacity assays (Figure 1B). ICR showed the highest TPC, 440.5 mg GAE/L, and tannin content, 360.7 mg/L, among the analyzed samples.

As shown in Figure 1B, the higher levels of tannins and selected hydroxycinnamic acids, including caftaric and caffeic acids, in Chardonnay wines may provide a chemical basis for their enhanced bioactivity [25,30]. This quantitative advantage was reflected in the antioxidant capacity results, with ICR showing the highest values in both the CUPRAC, 2.91 mmol TE/L, and DPPH, 1.66 mmol TE/L, assays. Interestingly, although CCR and CSB showed lower concentrations of bioactive compounds, the molar efficiency (ME) remained relatively stable across all samples, ranging from 6.48 to 6.70. This suggests that while the total quantity of phenolic compounds may vary depending on geographical origin, terroir, and cultivar, the relative antioxidant efficiency of the polyphenolic fraction remains broadly comparable among the analyzed wines. AE suggests also that antioxidant efficiency depends not only on the total amount of phenolics but also on their chemical structure, interactions and the overall wine matrix.

Ćorković et al. [2] reported that catechins, hydroxycinnamic acids and hydroxybenzoic acids are among the main phenolic groups responsible for the biological properties of white wines. In the present study, the high levels of gallic acid and catechin in ICR may partly explain its highest antioxidant activity. Gallic acid has strong reducing properties, while catechin can efficiently scavenge free radicals due to its hydroxyl groups. This agrees with Mitić et al. [31], who showed that white wines with higher phenolic content generally exhibit stronger antioxidant activity in assays such as DPPH. In CCR, the most characteristic feature was the highest caftaric acid concentration, 42.82 mg/L. This is consistent with literature data indicating that caftaric acid is one of the dominant hydroxycinnamic acids in white wines. Therefore, the elevated caftaric acid level in CCR suggests a distinct phenolic profile of Chilean Chardonnay compared with Israeli Chardonnay. Chardonnay wines, especially ICR, showed a more favorable bioactive profile than Sauvignon Blanc, as reflected by higher total phenolic content, tannin concentration and antioxidant capacity. Similar observations were reported by Kropek et al. [32] and Tița et al. [33], who emphasized that phenolic composition and antioxidant potential strongly depend on grape variety and wine-growing region.

Although the present Chardonnay samples, particularly ICR, showed higher total phenolic content, tannin concentration, and chemical antioxidant capacity than the analyzed Sauvignon Blanc samples, this pattern should not be regarded as a universal cultivar-related characteristic. Previous studies have reported considerable variability among Chardonnay and Sauvignon Blanc wines, and in some datasets Sauvignon Blanc or other white wine cultivars showed comparable or higher concentrations of selected phenolic compounds and antioxidant activity. Such apparently contradictory observations indicate that cultivar alone does not determine the phenolic or antioxidant profile of white wine. The differences observed between the Israeli and Chilean wines may reflect the combined influence of cultivar, geographical origin, climate, grape maturity, vinification procedures, extraction conditions, fermentation, ageing, and storage. For example, the relatively high gallic acid and catechin concentrations in ICR and the high caftaric acid concentration in CCR may result from differences in grape composition as well as producer-specific processing practices. Because only one producer from each country and one vintage were included, the effects of national origin cannot be separated from those of winery, production technology, and individual product formulation. The observed differences should therefore be interpreted as characteristics of the investigated commercial wines rather than as general differences between Israeli and Chilean wines [34,35,36,37].

### 3.2. FTIR Spectroscopic Characterization: Phytochemical Fingerprint and Antioxidant Capacity

**Figure 2:**
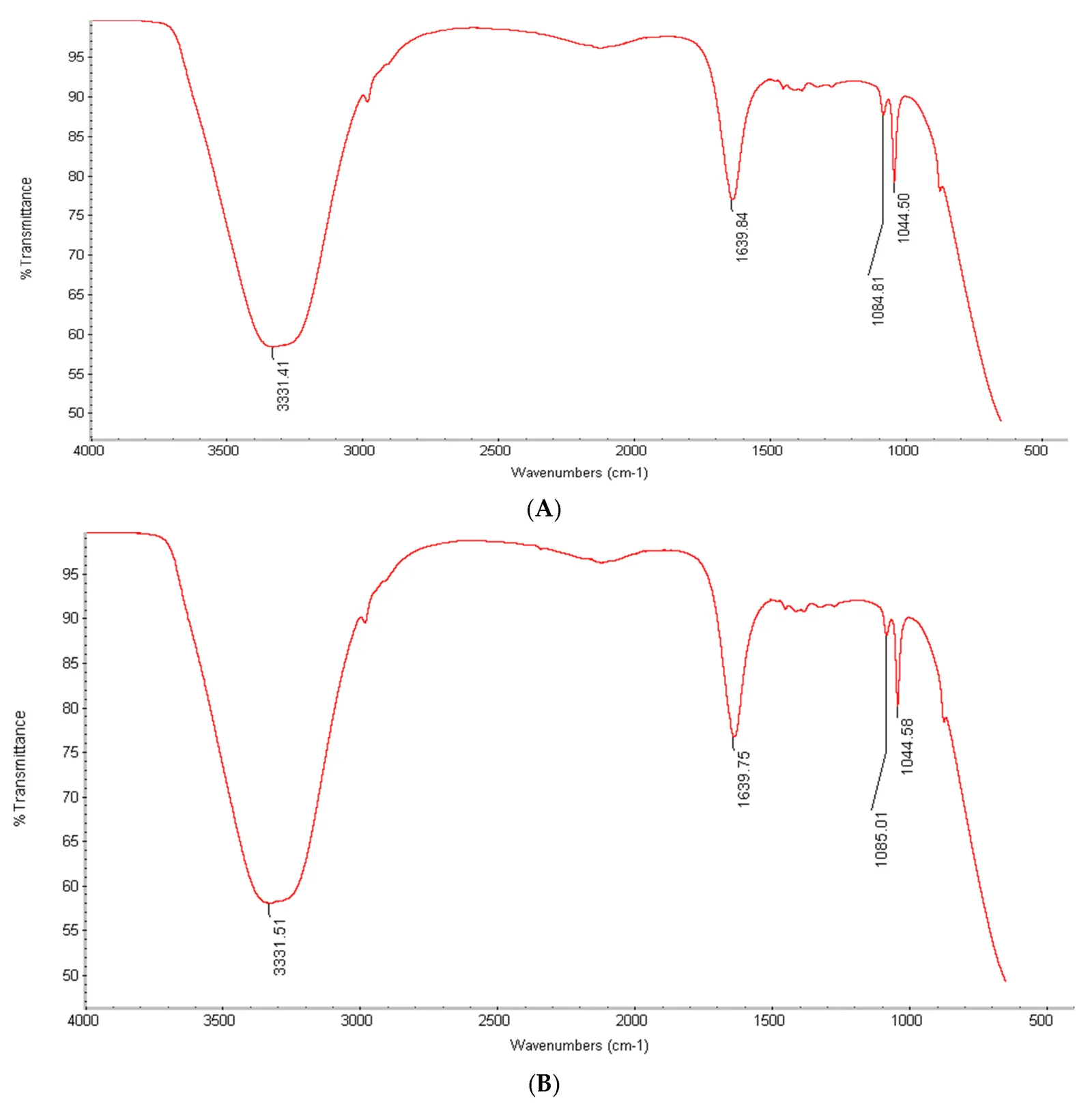
FTIR spectra of selected white wine samples (ISB (**A**) and ICR (**B**)) in the 4000–500 cm^−1^ region.

The FTIR spectra of the Israeli and Chilean white wines showed a consistent phenolic profile across all samples, characterized by two major absorption regions (Figure 2). All spectra exhibited a broad, intense band at 3333 cm^−1^, which confirms a high density of hydroxyl (–OH) groups. In the mid-IR fingerprint region (1800–900 cm^−1^), the wine extracts closely overlapped with the pure gallic acid and catechin standards. The main experimental peaks identified were at 1705 cm^−1^ and 1050 cm^−1^, showing carboxyl and ester peaks matching the gallic acid standard, confirming the presence of hydroxybenzoate structures. Aromatic skeletal vibrations at ~1610 cm^−1^, showed significant overlap with the catechin standard, indicating aromatic C=C stretching. A sharp peak at 1639 cm^−1^ represented carbonyl stretching from flavonoids. The intensity of this peak was noticeably higher in ICR samples compared to the Sauvignon Blanc samples, which directly supports our HPLC data showing higher phenolic content in Chardonnay. Distinct peaks at 1084 cm^−1^ and 1044 cm^−1^ corresponded to C–O and C–C stretching vibrations.

The Chilean cultivars (CCR and CSB) showed nearly identical spectral profiles and overlapping bands with the Israeli samples (ICR and ISB). Therefore, the extraction procedure successfully retained the core chemical features of these wine matrices across both regions. This comparative fingerprinting indicates that the ICR and ISB extracts contain functional groups with spectral characteristics similar to those of the analyzed phenolic standards. These structural similarities provide a physicochemical basis for interpreting the subsequent antioxidant and protein-binding results.

Our FTIR fingerprinting results confirm that the extraction method successfully preserved the active phenolic groups necessary for protein-binding studies without altering their chemical structures. The presence of specific bands at 3333 cm^−1^, 1705 cm^−1^, and 1639 cm^−1^ provides direct evidence that these white wines are rich in available hydroxyl and carbonyl groups. These findings are consistent with Scano [38], who demonstrated that the mid-infrared spectra of white wines contain reliable indicator bands for aromatic structures and non-flavonoid phenolic compounds. Similarly, Clarke et al. [39] showed that FTIR is a highly effective tool for identifying overlapping signals from phenolic acids and flavan-3-ols in grape-derived matrices. By mapping these specific functional groups, we establish the chemical basis for the subsequent protein-binding experiments. The abundance of these aromatic rings and active hydroxyl sites explains why these wine extracts can interact effectively with serum transport proteins.

### 3.3. Excitation–Emission Matrix Fluorescence and Binding Dynamics of Human Carrier Proteins

**Figure 3:**
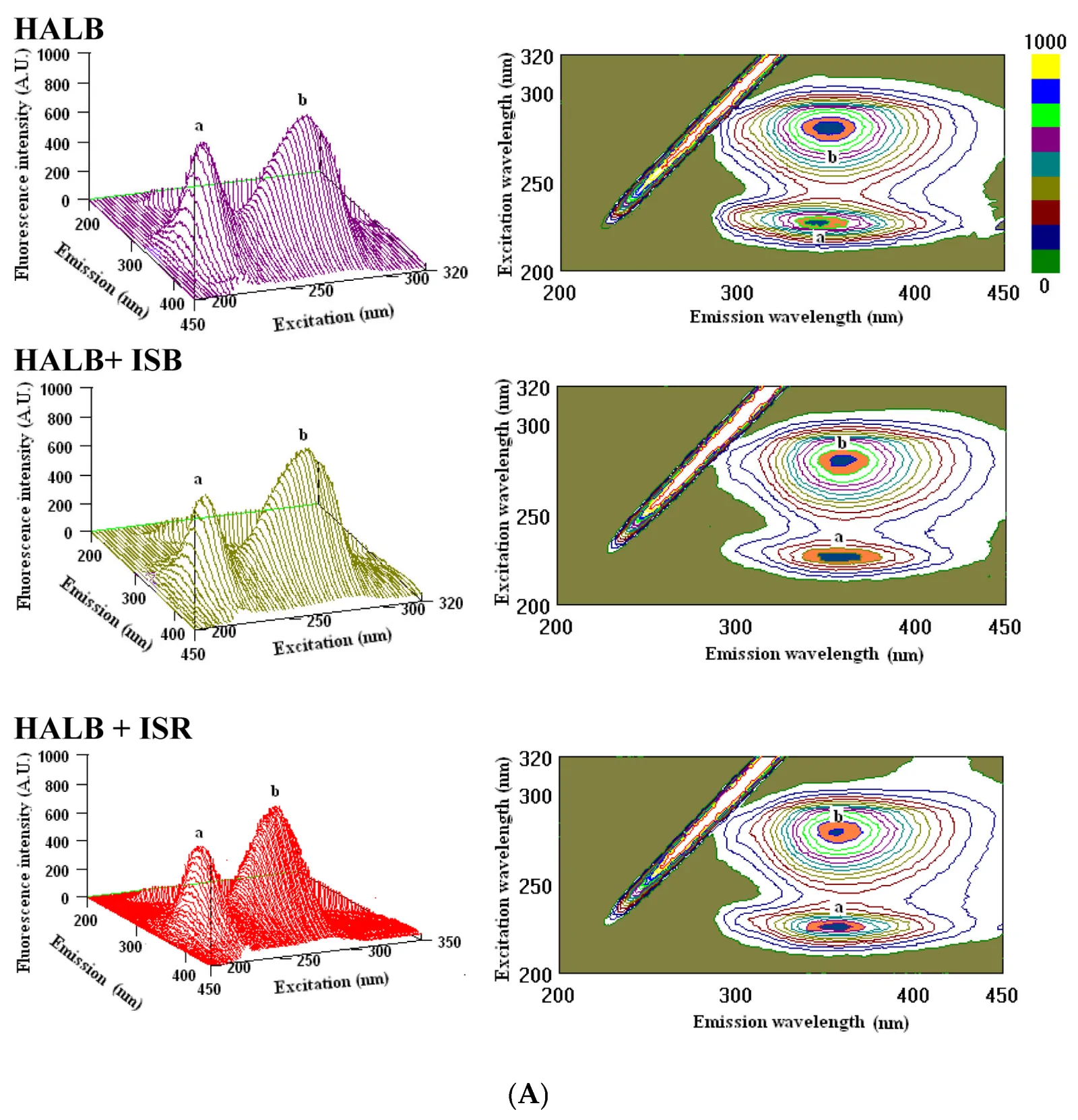

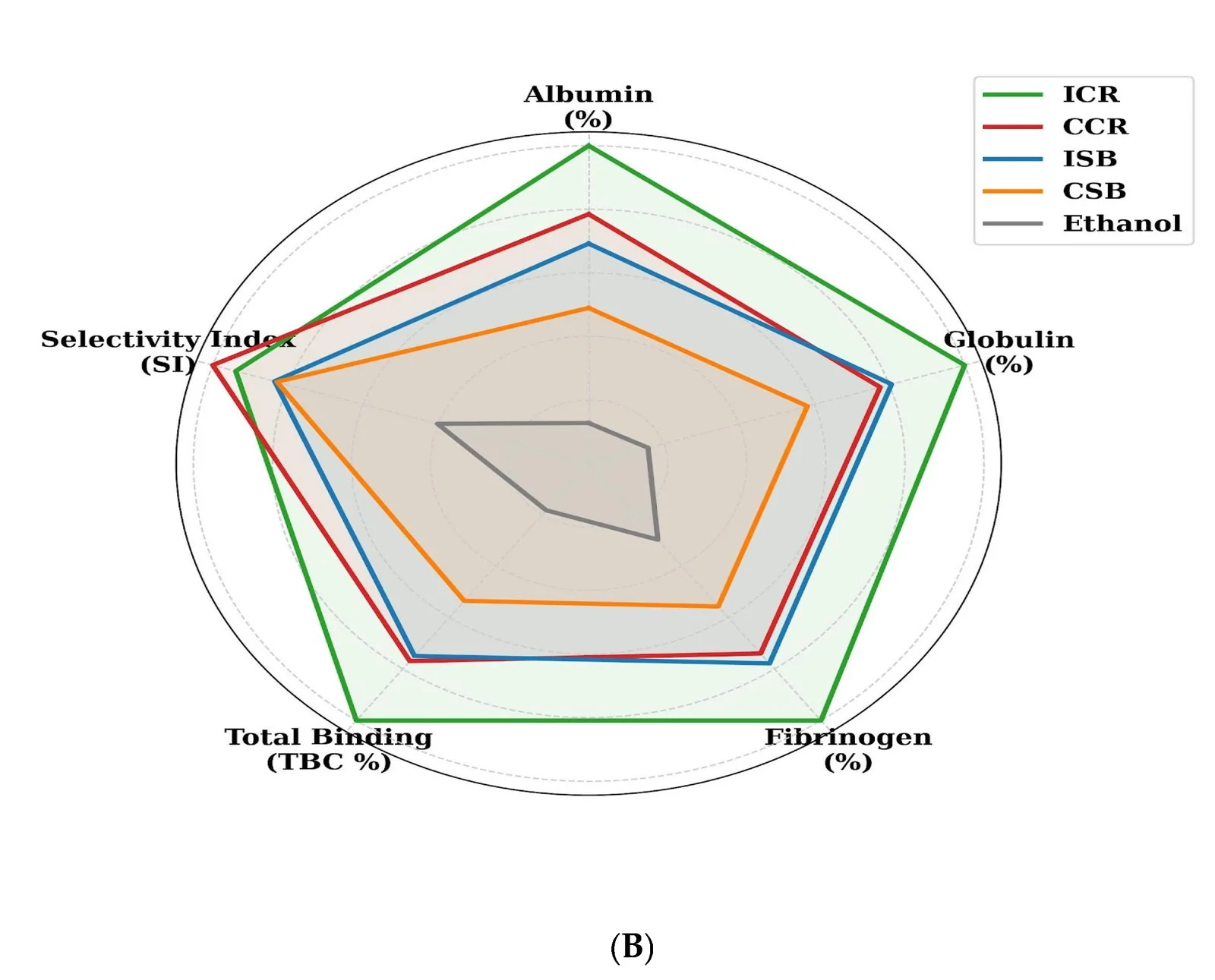
(**A**). Selected three-dimensional (3D) fluorescence spectra and their fluorometric profiles of: HALB (native human serum albumin with peak a of 643.05 A.U. and peak b of 920.10 A.U.); HALB + ISB (native human serum albumin after interaction with polyphenols extracted from Israeli Sauvignon Blanc with peak a of 586.16 A.U. and peak b of 876.40 A. U.); HALB + ICR (native human serum albumin after interaction with polyphenols extracted from Israeli Chardonnay with peak a of 552.85 A.U. and peak b of 865.79 A.U.). (**B**) Comparative binding profile of Israeli and Chilean white wines toward human carrier proteins. Radar plot showing the relative binding capacity of wine-derived polyphenols to albumin, globulin, and fibrinogen, together with total binding capacity (TBC%) and selectivity index (SI). The corresponding numerical values used to construct the radar plot are provided in Table 1. ICR, Israeli Chardonnay; CCR, Chilean Chardonnay; ISB, Israeli Sauvignon Blanc; CSB, Chilean Sauvignon Blanc. Ethanol was used as a control. Higher values indicate stronger protein-binding affinity and/or greater selectivity.

Three-dimensional excitation–emission matrix (EEM) fluorescence analysis revealed differences in the interaction profiles of the analyzed wine extracts with human serum proteins (Figure 3A). Native human serum albumin (HALB) exhibited two prominent fluorescence features: Peak a at λex/λem = 225/340 nm and Peak b at λex/λem = 280/340 nm. Following the addition of the isolated white wine polyphenol extracts, fluorescence intensity decreased at both peak coordinates without substantial shifts in the emission maxima. The greatest decrease was observed for the ICR extract, followed by ISB. These changes indicate modification of the microenvironment surrounding the intrinsic fluorescent residues of HALB under the applied in vitro conditions.

The comparative binding profiles toward HALB, HGLO, and HFB are summarized in Figure 3B and Table 1. The total binding capacities followed the order ICR (46.4%) > CCR (35.7%) > ISB (34.8%) > CSB (24.9%) > ethanol (8.5%). ICR showed the highest albumin-binding capacity, reaching 19.7%, whereas CSB showed the lowest value among the wine samples, 9.6%.

The Selectivity Index ranged from 1.36 to 1.64 for the wine extracts, indicating a higher apparent interaction with albumin than with globulin or fibrinogen. CCR showed the highest SI value, 1.64, whereas the ethanol control showed an SI below 1.0, at 0.66. The overall apparent binding tendency was HALB > HGLO > HFB. ISB and CCR showed statistically comparable total binding capacities despite differences in cultivar and geographical origin.

The decrease in HALB fluorescence intensity observed after the addition of the wine extracts is consistent with an interaction between wine-derived phenolics and the microenvironment surrounding intrinsic tryptophan and tyrosine residues. The absence of substantial shifts in the emission maxima suggests that the interaction affected local fluorophore accessibility without causing extensive changes in the overall polarity of the fluorescent microenvironment. Nevertheless, fluorescence quenching alone cannot establish the exact binding site or fully distinguish between static, dynamic, and mixed quenching contributions. The higher apparent binding observed for the Chardonnay extracts, particularly ICR, may be related to their greater contents of catechin, gallic acid, and other hydroxyl-rich aromatic compounds. These molecules can participate in hydrogen bonding, hydrophobic interactions, and aromatic stacking with albumin residues. However, the observed binding profile cannot be attributed to individual compounds alone because wine constitutes a complex chemical matrix [40]. Additive, antagonistic, competitive, or other matrix-dependent interactions among multiple phenolic constituents may also contribute to the measured fluorescence response. The substantially weaker response of the ethanol control indicates that the observed in vitro protein interactions were primarily associated with the non-ethanolic wine matrix rather than with ethanol alone. This finding does not demonstrate that ethanol has no influence under physiological conditions, nor does it confirm that wine phenolics form stable transport complexes in the circulation. Whether the observed interactions influence the persistence, distribution, or metabolism of these compounds in vivo requires pharmacokinetic investigation at physiologically achievable concentrations and in the presence of circulating metabolites and competing endogenous ligands.

The preference for HALB over HGLO and HFB is compatible with the known ligand-binding properties of albumin and with the complementary docking predictions involving Sudlow site I [41]. However, the EEM results do not directly identify Sudlow site I as the exclusive binding region. The apparent preference for albumin may also reflect differences in protein structure, accessibility of fluorescent amino acid residues, molecular size, and the number and heterogeneity of potential ligand-binding regions among the analyzed proteins [42]. Such selective binding is consistent with the docking results and identifies albumin as the preferred molecular target among the proteins tested. However, it does not demonstrate systemic transport in vivo [29]. The preferential interaction of wine-derived phenolics with HALB may also have pharmacokinetic implications because several drugs bind within Sudlow sites I and II. In principle, compounds occupying overlapping or allosterically connected albumin-binding regions may alter the unbound fraction of co-administered drugs. However, such displacement is highly dependent on the structure and circulating concentration of the phenolic compound and its metabolites, as well as on the binding affinity, therapeutic index, distribution, and elimination of the drug. Although the present docking results indicate that selected wine phenolics may interact with residues located within Sudlow site I, this study did not directly assess competition with any pharmaceutical ligand. Therefore, potential drug displacement should be regarded as a hypothesis requiring confirmation in dedicated competitive binding assays and subsequent pharmacokinetic studies [43,44]. The similar total binding capacities observed for ISB and CCR, despite differences in cultivar and geographical origin, further indicate that total phenolic concentration alone does not fully explain the interaction profile. Differences in phenolic composition, molecular polarity, steric accessibility, and interactions among matrix constituents may produce comparable net binding responses. This provides an alternative explanation to a simple concentration-dependent mechanism. The interpretation of these findings is limited by the use of isolated proteins and wine extracts under controlled in vitro conditions. The experimental model does not reproduce gastrointestinal digestion, intestinal absorption, phase II metabolism, plasma protein competition, or the substantially lower concentrations of parent polyphenols that may occur in vivo. Consequently, the present results provide mechanistic evidence of protein–polyphenol interactions but should not be interpreted as direct proof of systemic transport, enhanced bioavailability, prolonged circulation, clinically significant drug displacement, or antioxidant protection in humans.

### 3.4. Thermodynamic Parameters and Stern–Volmer Analysis of Albumin–Phenolic Interactions

**Figure 4:**
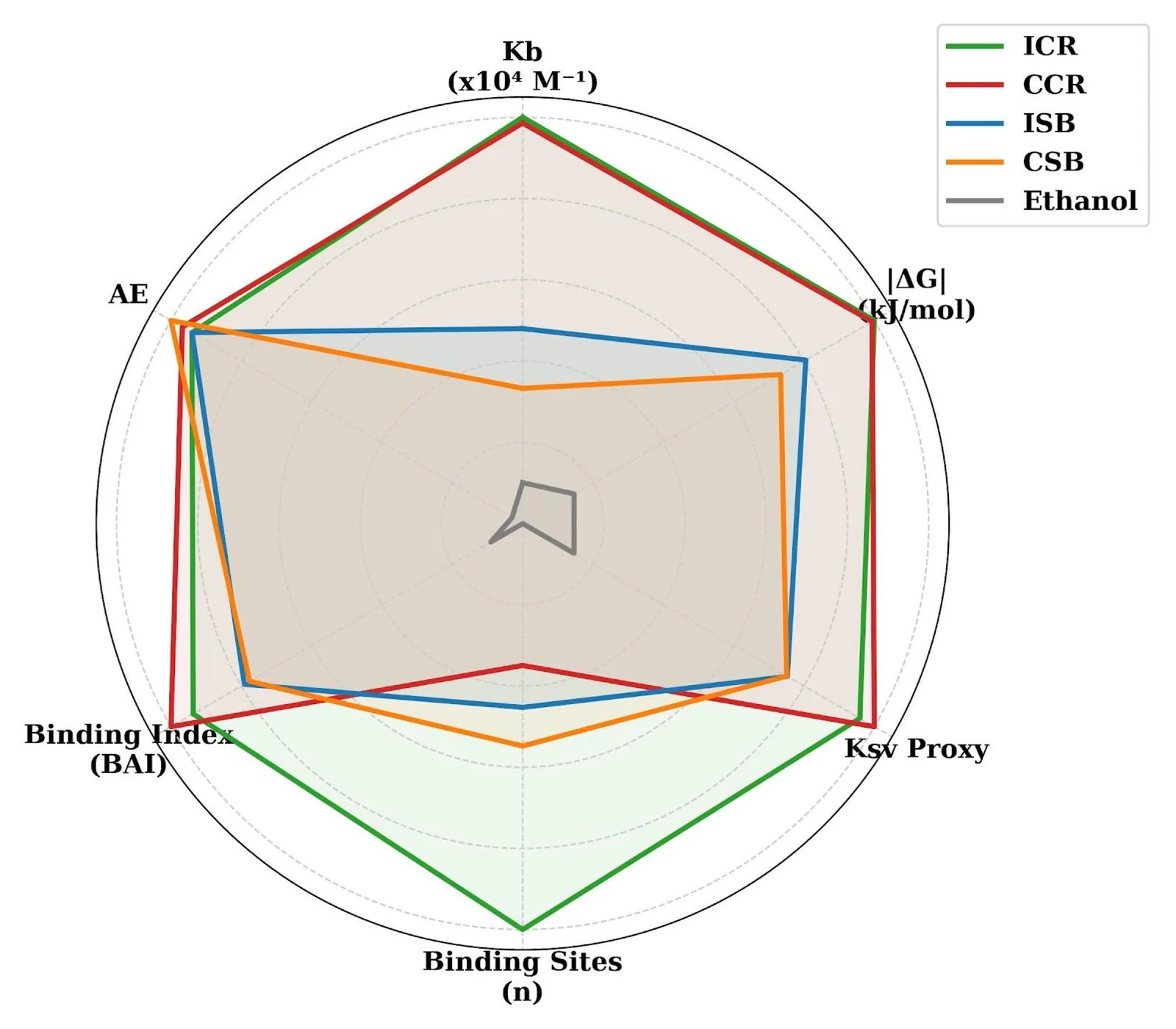
Thermodynamic and binding parameters of wine-derived polyphenols toward human serum albumin. Radar plot comparing the binding constant (Kb × 10^4^ M^−1^), Gibbs free energy change (ΔG, kJ/mol), Stern–Volmer quenching proxy (Ksv proxy), number of binding sites (n), binding affinity index (BAI), and antioxidant efficiency (AE) for Israeli and Chilean Chardonnay and Sauvignon Blanc wines. The corresponding numerical values used to construct the radar plot are provided in Supplementary Table S3. ICR, Israeli Chardonnay; CCR, Chilean Chardonnay; ISB, Israeli Sauvignon Blanc; CSB, Chilean Sauvignon Blanc. Ethanol was used as the control.

The docking analysis focused on human serum albumin because these proteins represent two functionally distinct but biologically relevant targets in the circulatory system. Human serum albumin is the major plasma transport protein and plays a central role in the binding, stabilization, and systemic distribution of endogenous and dietary bioactive compounds, including polyphenols. Therefore, analysis of albumin–phenolic interactions provides insight into the potential bioavailability and transport capacity of white wine phytochemicals. Figure 4 provides thermodynamic and fluorescence-derived evidence for the interaction between wine-derived polyphenols and human serum albumin, including Kb, ΔG, KSV, n, BAI, and AE values. For example, the binding affinity constant (Kb) for ICR was 8.44 × 10^4^ M^−1^, placing it in the same statistical group as the catechin and epicatechin standards, whereas CSB showed a lower Kb value of 2.81 × 10^4^ M^−1^. The negative calculated ΔG values indicate that complex formation was thermodynamically favorable under the applied experimental and computational conditions [29]. The statistical similarity between Chardonnay extracts and catechin or epicatechin standards indicates comparable apparent albumin-binding parameters under the applied assay conditions. This comparison should not be interpreted as evidence of equivalent biological activity or bioavailability in vivo [28]. The higher Stern–Volmer proxy (KSV) observed for Chardonnay samples indicates more efficient fluorescence quenching and suggests greater accessibility of their phytochemicals to albumin-binding regions [32]. The integrated analysis indicated high-efficiency targeting based on the combination of SI and KSV values. The relatively high SI and KSV values observed for Chardonnay suggest that its phytochemicals do not bind randomly but show preferential interaction with HALB, possibly involving hydrophobic binding pockets. The Kb values for Chardonnay wines, 8.44 and 8.32 × 10^4^ M^−1^, were statistically comparable to those of pure catechin, 8.12 × 10^4^ M^−1^, and epicatechin, 8.91 × 10^4^ M^−1^. These results support the hypothesis that the stronger albumin-binding and fluorescence-quenching behavior of the Chardonnay extracts is partly associated with their flavan-3-ol content. These flavonoids may act as structural anchors that contribute to the superior docking performance and fluorescence quenching observed in Chardonnay samples. The higher KSV values for Chardonnay, 0.00055–0.00058, compared with Sauvignon Blanc, approximately 0.00043, further support the quenching data and indicate a more efficient interaction pathway between Chardonnay phytochemicals and albumin-binding sites. From the “quality versus quantity” perspective, the Binding-to-Antioxidant Index (BAI = Kb (Binding Constant)/Antioxidant Efficiency (AE)) shows that even when wines display similar antioxidant efficiency (AE, approximately 0.003), Chardonnay, with a BAI of 12.69, is approximately 28% more effective in protein loading than Sauvignon Blanc, with a BAI of 9.85. This may indicate a more stable systemic antioxidant-binding profile. Among the tested wines, Chilean Chardonnay (CCR) emerged as one of the strongest performers, showing the highest selectivity index, 1.64, and binding index, 12.69. This effect may be related to terroir-dependent differences in phytochemical composition and bioactivity. These findings suggest that Chardonnay wines, particularly CCR and ICR, possess a more favorable albumin-binding profile than Sauvignon Blanc wines, and that this behavior is mainly associated with their phenolic composition rather than ethanol content alone.

### 3.5. Comparative Binding Affinity of Phenolic Monomers Toward Human Serum Albumin

**Figure 5:**
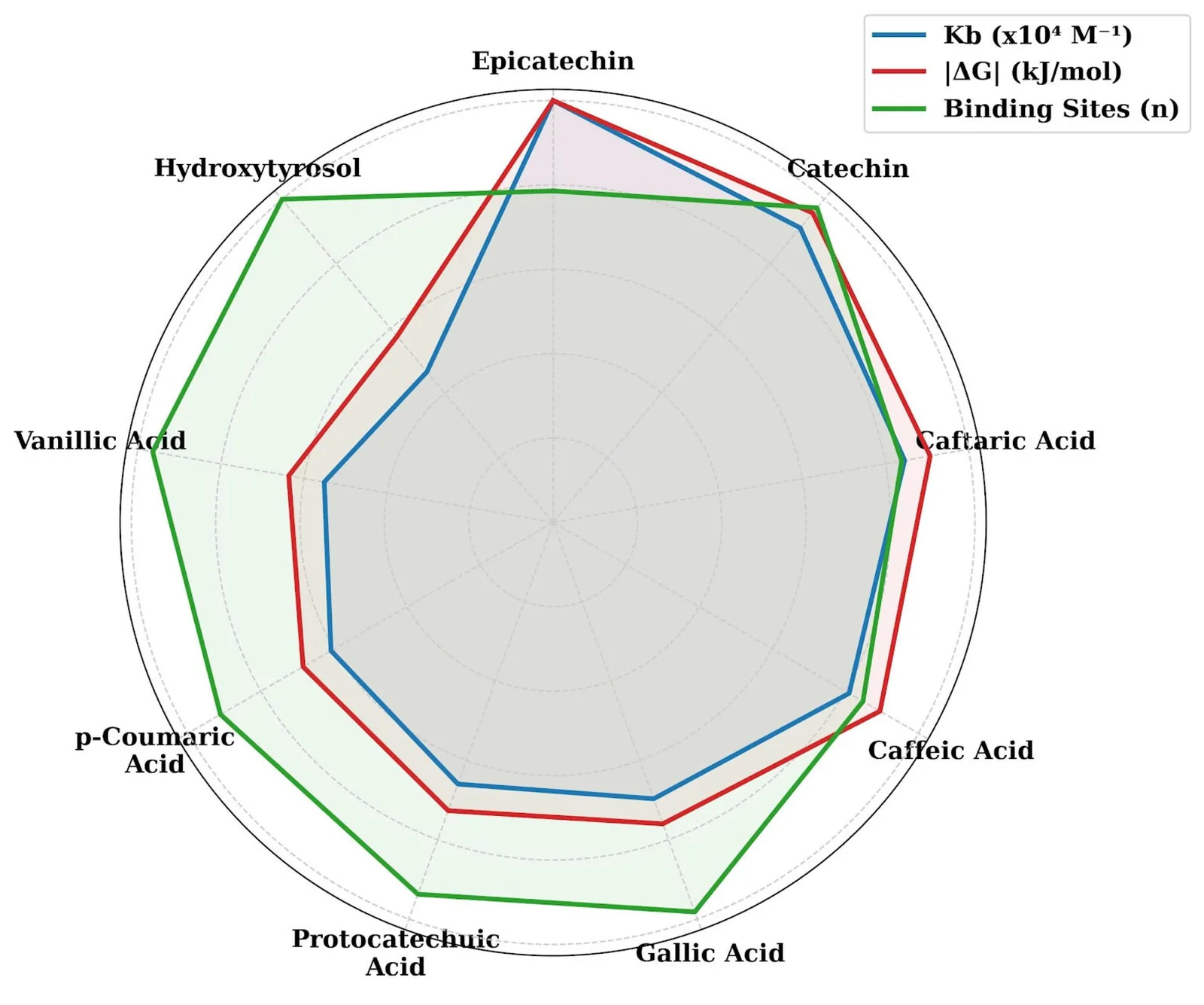
Comparative affinity of selected phenolic monomers toward human serum albumin. Radar plot showing the normalized binding constant (Kb × 10^4^ M^−1^), absolute Gibbs free energy change (ΔG, kJ/mol), and number of binding sites (n) for selected wine-related phenolic standards, including epicatechin, catechin, caftaric acid, caffeic acid, gallic acid, protocatechuic acid, *p*-coumaric acid, vanillic acid, and hydroxytyrosol. The corresponding numerical values used to construct the radar plot are provided in Supplementary Table S4. Higher Kb and ΔG values indicate stronger and more thermodynamically favorable interactions with human serum albumin, while n represents the estimated number of binding sites.

The analysis of pure phenolic standards (Figure 5) confirms that flavonoids such as epicatechin and catechin exhibited the highest binding affinity for HALB, with Kb values exceeding 8.0 × 10^4^ M^−1^. This strong affinity is consistent with the superior binding performance of Israeli Chardonnay (ICR), as shown in Figure 3, and is supported by FTIR fingerprinting (Figure 2), which indicated the presence of aromatic and hydroxyl-rich compounds in these wines. The spontaneous nature of the binding process was further confirmed by the negative ΔG values observed for all standards. Interestingly, although gallic acid showed moderate affinity when tested individually, Kb = 6.21 × 10^4^ M^−1^, its presence in the wine matrix may contribute to the high total binding capacity (TBC) through additive electrostatic and hydrogen-bonding interactions.

These comparative data suggest that the ability of wine-derived compounds to interact with human carrier proteins, and potentially influence their structural properties, is closely related to the specific phytochemical composition of each wine. In particular, flavan-3-ols such as catechin and epicatechin appear to be important contributors to the enhanced albumin-binding profile observed for Chardonnay samples [25,26,27].

Conversely, Sauvignon Blanc showed greater similarity to caftaric acid and other hydroxycinnamate-related standards, such as caffeic and gallic acids, which was reflected in its lower overall binding affinity. While pure standards displayed binding-site values close to 1.0, suggesting a predominantly 1:1 molecular interaction, the wine samples showed lower n values, ranging from 0.26 to 0.69. This may indicate that, in a complex wine matrix, molecular crowding, competition, or synergistic effects occur, where multiple phenolic compounds may interact with or compete for the same protein-binding region. Although phenolic acid standards exhibited measurable affinity toward HALB, their thermodynamic profiles were generally less favorable than those observed for flavonoid standards. This may help explain why ISB and CSB showed lower thermodynamic stability in protein-binding complexes compared with Chardonnay samples. Furthermore, the significant difference between the flavonoid group and the phenolic acid group, supports the distinction between the binding profiles of Chardonnay and Sauvignon Blanc wines. The high similarity between ICR and epicatechin suggests that epicatechin may be one of the key contributors to protein binding in white wine. The fact that ICR showed stronger Kb and ΔG-related parameters than catechin alone suggests that the natural wine matrix may provide a synergistic effect, enhancing binding beyond the activity of a single isolated compound. The presence of gallic acid and hydroxytyrosol in Israeli Chardonnay may further contribute to a more complex docking environment. Although hydroxytyrosol displayed a lower individual Kb value, 4.15 × 10^4^ M^−1^, its coexistence with gallic acid and catechin may contribute to the more favorable spontaneous binding profile observed for ICR, ΔG = −3.12 kJ/mol [45,46,47,48].

### 3.6. Predicted Secondary Structure Shifts in HALB and Ethanol Control

The binding of wine-derived polyphenols induced measurable conformational changes in the predicted secondary structure of HALB. By monitoring the transition from α-helix to β-sheet and random coil structures, it was possible to estimate the structural impact of the phytochemical fingerprints identified in the previous sections. Among the tested samples, ICR and the epicatechin standard showed the most pronounced structural effects. Table 2 presents the predicted changes in α-helix preservation and β-sheet formation. Structural stability was associated with α-helix preservation above 60%, while β-sheet formation remained below 1.0%. These results suggest that the wine polyphenols, particularly those present in Chardonnay, may help maintain the native-like α-helical structure of HALB while limiting the formation of β-sheet-rich conformations. A key finding in Table 2 is the maintenance of α-helix integrity above 60% in the Chardonnay–HALB complexes [15]. By occupying high-affinity binding pockets, these phytochemicals may contribute to structural stabilization of HALB and reduce the tendency toward α-helix-to-β-sheet transition [21]. This effect is relevant because increased β-sheet formation is often associated with protein misfolding and aggregation processes [14,22]. The native HALB structure, with an α-helix content of approximately 67.0%, was used as the baseline control.

The predicted shift to 62.1% α-helix for the Chardonnay–HALB complex can be interpreted as a functional docking-related conformational adjustment rather than protein denaturation. The predicted β-sheet content remained below 1.0%, suggesting that the modeled complexes did not involve extensive conversion of the native α-helical structure into β-sheet-rich conformations. As shown in Table 2, at least 60% of the protein structure remained in the stable α-helical form across all tested samples. These calculations were based on the relationship between the binding constant (Kb) and the predicted thermodynamic displacement of the protein backbone. Importantly, even in the presence of high-affinity docking, particularly for Chardonnay samples, the α-helix content remained above 60%. This indicates that HALB did not undergo major structural disruption or loss of its native-like conformation. Instead, the protein retained its potential function as a transport carrier, suggesting that the observed conformational changes may reflect ligand accommodation rather than denaturation.

The Binding-to-Antioxidant Index (Figure 4) further suggests that the Chardonnay–protein complex has a more favorable structural-binding profile than the Sauvignon Blanc equivalent [19]. Predicted α-helix shift calculations were performed using the established baseline of 67.0% α-helix for native HALB. The α-helix content was reduced after the addition of Israeli wine extracts, with ICR showing a predicted decrease to 62.1%. This shift was similar to that observed for pure epicatechin, 61.8%, and catechin, 62.5%, suggesting that these monomers may be major contributors to the conformational changes observed. The 4–5% α-helix shift observed for Chardonnay should not be interpreted as structural damage. Rather, it may reflect a moderate rearrangement of HALB subdomains associated with ligand binding. Such a change suggests that HALB can accommodate a high-affinity antioxidant load while maintaining its overall structural integrity. This predicted conformational pattern is compatible with ligand accommodation without extensive disruption of the native-like albumin structure. Its consequences for ligand stability or transport in vivo remain unknown.

The ethanol control, which showed 66.8% α-helix, is particularly important for interpretation. Ethanol caused only a negligible shift of 0.2% compared with native HALB, indicating that alcohol alone had minimal influence on the predicted secondary structure of the protein. This supports the conclusion that the observed protein-remodeling effects are mainly associated with the polyphenolic matrix rather than the ethanol fraction. The predicted interaction of wine-derived polyphenols with albumin-binding regions, including Sudlow site I, is consistent with the FTIR fingerprint and binding data. These findings suggest that the spontaneous docking of polyphenols may induce limited, controlled conformational adjustments in HALB, facilitating ligand accommodation while preserving the protein’s carrier function.

### 3.7. Mechanistic Interpretation, Study Limitations, and Hypotheses for Future Research

In many pathological conditions, protein unfolding and increased β-sheet formation are associated with aggregation processes and inflammatory responses. The predicted β-sheet contribution remained below 1% in all modeled complexes. Within the limitations of the computational analysis, this result suggests that ligand binding was not associated with extensive β-sheet formation. However, the present study did not evaluate protein aggregation, inflammatory signaling, or anti-inflammatory activity. Therefore, no conclusions regarding structural protection or anti-inflammatory efficacy can be drawn from these data.

The slight increase in random coil/turn structures, from 33.0% to 37.1% in Chardonnay, indicates that the protein becomes more flexible around the binding site. This “induced fit” may allow albumin to accommodate wine-derived molecules and potentially protect them during transport, explaining the flexibility advantage associated with random coil formation.

Chardonnay, particularly ICR, produced a structural shift similar to that observed for epicatechin. This supports the hypothesis that flavan-3-ols, including catechin and epicatechin, are important contributors to the protein-binding and structural effects of Chardonnay wine. However, these findings should be interpreted as in vitro mechanistic evidence rather than direct proof of equivalent health benefits to purified flavonoid supplementation.

The present findings provide a mechanistic basis for interpreting the interactions between selected white wine phytochemicals and human serum proteins under controlled in vitro and in silico conditions [1,3,49,50,51,52]. The fluorescence-quenching and binding results are consistent with complex formation between wine-derived phenolics and the analyzed serum proteins, particularly human serum albumin. However, whether comparable complexes form at physiologically relevant concentrations and influence ligand stability, circulating half-life, distribution, or metabolism remains to be established. Hydroxycinnamic acids, including caftaric and caffeic acids, contributed to the phenolic profiles and antioxidant capacity of the analyzed wines. Their ability to participate in electron- or hydrogen-transfer reactions provides a plausible chemical explanation for the activity observed in the DPPH and CUPRAC assays. Nevertheless, these chemical antioxidant assays do not demonstrate inhibition of lipid oxidation, arterial inflammation, or oxidative stress in biological systems. Such effects require confirmation using cell-based lipid-peroxidation and oxidative stress models.

The calculated negative ΔG values indicate that the modeled albumin–phenolic interactions were thermodynamically favorable under the applied experimental and computational conditions. These parameters do not establish whether equivalent interactions occur after wine consumption, because gastrointestinal transformation, intestinal absorption, polyphenol metabolism, physiologically achievable plasma concentrations, and competition with endogenous and pharmaceutical ligands were not evaluated. Although white and red wines differ in their phenolic composition and the relative proportions of monomeric and polymeric compounds, the present study did not directly compare their absorption, metabolism, or ability to cross biological barriers. Therefore, the measured antioxidant efficiency and albumin-binding parameters should be interpreted solely as comparative physicochemical characteristics of the investigated white wine samples. Their physiological and nutritional relevance requires further evaluation in digestion models, cellular systems, pharmacokinetic studies, and ultimately animal or human investigations.

An important limitation of this study is the restricted number of wine products. The four selected wines represent specific commercial examples of Chardonnay and Sauvignon Blanc from Israel and Chile and should not be considered representative of the full diversity of these cultivars or national wine production. Although five bottles of each wine were obtained from different retail locations to improve product-level reliability and reduce the influence of individual-bottle variability, this replication does not compensate for the limited number of producers, labels, vintages, and geographical regions. Consequently, the observed differences should be interpreted as applying to the analyzed products rather than generalized to all Israeli or Chilean Chardonnay and Sauvignon Blanc wines. Future studies should include a larger number of producers, production batches, vintages, and wine-growing regions.

## 4. Conclusions

This study demonstrated that phenolic-rich extracts from the analyzed white wines interact with human serum albumin, gamma-globulin, and fibrinogen under controlled in vitro conditions, with the strongest apparent interaction observed for albumin. Fluorescence quenching and three-dimensional excitation–emission analysis indicated changes in the microenvironment surrounding albumin fluorescent residues, while molecular docking provided a complementary structural model of possible ligand-binding orientations. Chardonnay samples generally showed higher apparent binding parameters and antioxidant capacity than Sauvignon Blanc samples, whereas ethanol alone produced substantially weaker effects. These findings provide physicochemical and mechanistic evidence of protein–polyphenol interactions but do not demonstrate systemic bioavailability, pharmacokinetic effects, or health benefits in vivo. Further studies incorporating simulated digestion, polyphenol metabolites, physiologically relevant concentrations, competitive binding assays, cellular models, and ultimately animal or human studies are required to establish the biological significance of these interactions.

## Figures and Tables

**Table 1:** Binding capacity of wine-derived polyphenols toward human carrier proteins and selectivity index.

Sample	Albumin (%)	Globulin (%)	Fibrinogen (%)	Total Binding (TBC%)	Selectivity Index (SI)
ICR	19.7 ± 0.8 ^a^	13.9 ± 0.6 ^a^	12.8 ± 0.5 ^a^	46.4 ± 1.1 ^a^	1.54 ^a^
CCR	15.5 ± 0.6 ^b^	10.8 ± 0.4 ^b^	9.4 ± 0.4 ^b^	35.7 ± 0.8 ^b^	1.64 ^a^
ISB	13.6 ± 0.5 ^b^	11.2 ± 0.4 ^b^	9.9 ± 0.3 ^b^	34.8 ± 0.7 ^b^	1.37 ^b^
CSB	9.6 ± 0.3 ^d^	8.1 ± 0.2 ^c^	7.1 ± 0.2 ^c^	24.9 ± 0.4 ^c^	1.36 ^b^
Ethanol	2.5 ± 0.1 ^e^	2.2 ± 0.2 ^e^	3.8 ± 0.3 ^e^	8.5 ± 0.8 ^e^	0.66 ^e^

Values are expressed as mean ± SD. Different superscript letters within the same column indicate statistically significant differences between samples (*p* < 0.05). Samples sharing the same letter within a column are not significantly different. ICR, Israeli Chardonnay; CCR, Chilean Chardonnay; ISB, Israeli Sauvignon Blanc; CSB, Chilean Sauvignon Blanc; TBC, total binding capacity, calculated as the sum of albumin, globulin, and fibrinogen binding percentages; SI, selectivity index, calculated as the ratio of albumin binding to the average binding toward globulin and fibrinogen. Ethanol was used as the control.

**Table 2:** Predicted Secondary Structure Shifts in HALB.

Sample/Standard	α-Helix (%)	β-Sheet (%)	Random Coil/Turn (%)
Native Protein	67.0	0.0	33.0
ICR	62.1	0.8	37.1
CCR	62.3	0.7	37.0
ISB	64.8	0.3	34.9
CSB	65.5	0.2	34.3
Epicatechin	61.8	0.9	37.3
Catechin	62.5	0.7	36.8
Caftaric Acid	62.9	0.6	36.5
Caffeic Acid	63.2	0.6	36.2
Gallic Acid	63.8	0.5	35.7
Protocatechuic Acid	64.1	0.4	35.5
*p*-Coumaric Acid	64.4	0.4	35.2
Vanillic Acid	64.7	0.3	35.0
Hydroxytyrosol	65.1	0.2	34.7
Ethanol	66.8	0.0	33.2

Abbreviations: ICR, Israeli Chardonnay; CCR, Chilean Chardonnay; ISB, Israeli Sauvignon Blanc; CSB, Chilean Sauvignon Blanc; HALB, Human Serum Albumin.

## Data Availability

The original contributions presented in this study are included in the article/Supplementary Material. Further inquiries can be directed to the corresponding author(s).

## Supplementary Materials

The following supporting information can be downloaded at: https://www.mdpi.com/article/10.3390/biom16081153/s1, Supplementary Materials S1. Equations and Calculation Procedures for Protein-Binding, Fluorescence Quenching, and Thermodynamic Analysis; Supplementary Materials S2. Calculation of antioxidant efficiency and molar efficiency; Table S1. Concentrations of selected phenolic compounds in Israeli and Chilean white wines used to construct the radar plot presented in Figure 1A; Table S2. Total phenolic content, tannin content, antioxidant capacity, and molar efficiency of Israeli and Chilean white wines used to construct the radar plot presented in Figure 1B; Table S3. Thermodynamic, fluorescence-quenching, and binding parameters of wine-derived polyphenols toward human serum albumin used to construct Figure 4; Table S4. Binding and thermodynamic parameters of selected wine-related phenolic standards toward human serum albumin used to construct the radar plot.

## Abbreviations

The following abbreviations are used in this manuscript:

ABV	Alcohol by volume
AE	Antioxidant efficiency
ANOVA	Analysis of variance
ATR	Attenuated total reflectance
BAI	Binding-to-Antioxidant Index/Binding Affinity Index
CAD	Coronary artery disease
CCR	Chilean Chardonnay
CSB	Chilean Sauvignon Blanc
CUPRAC	Cupric Reducing Antioxidant Capacity
DPPH	2,2-diphenyl-1-picrylhydrazyl
DSSP	Define Secondary Structure of Proteins
EEM	Excitation–Emission Matrix
FTIR	Fourier-Transform Infrared Spectroscopy
GAE	Gallic Acid Equivalents
HALB	Human Serum Albumin
HFB	Human Fibrinogen
HGLO	Human Gamma-Globulin
HPLC	High-Performance Liquid Chromatography
ICR	Israeli Chardonnay
ISB	Israeli Sauvignon Blanc
Kb	Binding constant
KSV	Stern–Volmer quenching constant
LDL	Low-Density Lipoprotein
PDB	Protein Data Bank
SI	Selectivity Index
TBC	Total Binding Capacity
TE	Trolox Equivalents
TPC	Total Phenolic Content
TNs	Total Tannins
ΔG	Gibbs Free Energy Change
2D-FL	Two-Dimensional Fluorescence Spectroscopy
n	number of binding sites
