# Supplementary material for: Molecular Interactions and Antioxidant Properties of White Wine Phytochemicals: A Mechanistic Study of Serum Protein Binding
Source: Biomolecules. 2026 Aug 7;16(8):1153. doi: 10.3390/biom16081153 (PMC13510347; doi:10.3390/biom16081153)
Supplement: Supplementary file 1 [file biomolecules-16-01153-s001.zip › biomolecules-4454075-supplementary.pdf]

Supplementary Materials S1. Equations and Calculation Procedures for Protein-Binding, Fluorescence Quenching, and Thermodynamic Analysis

The selectivity index (SI) was calculated to compare the relative affinity of wine polyphenols toward albumin and globulin according to the following equation:

SI = albumin-binding capacity (%) / globulin-binding capacity (%)

This parameter was used to evaluate the preferential interaction of wine phenolics with HALB compared with HGLO.

The fluorescence quenching efficiency of wine phenolics toward HALB was evaluated using the Stern–Volmer equation:

$$F_0/F = 1 + KSV[Q]$$

where F_0 is the fluorescence intensity of the native protein in the absence of the quencher, F is the fluorescence intensity in the presence of the wine sample, [Q] is the quencher concentration, and KSV is the Stern–Volmer quenching constant. KSV was determined from the slope of the linear plot of F_0/F versus [Q]. For individual measurements, the apparent Stern–Volmer value was calculated as:

$$KSV = (F_0/F - 1)/[Q]$$

or equivalently:

$$KSV = (F_0 - F)/(F \times [Q])$$

The KSV value was used as an indicator of quenching efficiency and the accessibility of wine phenolics to the fluorescent residues of the protein. The interaction site suggested by fluorescence analysis was interpreted together with molecular docking results, particularly in relation to Sudlow site I of human serum albumin.

To determine the binding constant and the number of binding sites, fluorescence quenching data were analyzed using the double-logarithmic equation:

$$\log[(F_0 - F)/F] = \log Kb + n \log[Q]$$

where Kb is the binding constant, representing the stability of the protein–ligand complex, and n is the number of binding sites. The values of Kb and n were obtained from the intercept and slope, respectively, of the plot of $\log[(F_0 - F)/F]$ versus log[Q] [22].

The Gibbs free energy change, ΔG, was calculated to evaluate the spontaneity of the binding process according to the following equation:

$$\Delta G = -RT \ln Kb$$

where R is the universal gas constant, 8.314 J/mol·K, T is the absolute temperature in kelvin, and Kb is the binding constant. Negative ΔG values indicate that the interaction between wine phenolics and the protein occurs spontaneously [23, 24].

Supplementary Materials S2. Calculation of antioxidant efficiency and molar efficiency

S2.1. Antioxidant efficiency

Antioxidant efficiency (AE) was calculated as the ratio of DPPH antioxidant capacity to total phenolic content:

$$AE = \frac{\text{DPPH}}{\text{TPC}}$$

where DPPH is expressed as mmol TE/L and TPC as mg GAE/L.

S2.2. Molar efficiency

Molar efficiency (ME) was calculated as the ratio of CUPRAC antioxidant capacity to total phenolic content, multiplied by 1000 to facilitate numerical presentation:

$$ME = \frac{\text{CUPRAC}}{\text{TPC}} \times 1000$$

where CUPRAC is expressed as mmol TE/L and TPC as mg GAE/L. The factor of 1000 was used solely as a scaling factor.

Table S1. Concentrations of selected phenolic compounds in Israeli and Chilean white wines used to construct the radar plot presented in Figure 1A.

**Compound**	**ICR**	**CCR**	**ISB**	**CSB**
Phenolic Acids				
**Caftaric acid**	36.71 ± 1.35^{b}	42.82 ± 1.61^{a}	30.18 ± 1.15^{c}	32.43 ± 1.22^{c}
**Caffeic acid**	3.78 ± 0.14^{b}	2.81 ± 0.11^{c}	4.75 ± 0.18^{a}	3.24 ± 0.12^{bc}
**Gallic acid**	7.21 ± 0.28^{a}	5.22 ± 0.20^{b}	4.18 ± 0.16^{c}	2.82 ± 0.11^{d}
**p-Coumaric acid**	2.41 ± 0.09^{a}	1.94 ± 0.07^{b}	1.75 ± 0.07^{bc}	1.18 ± 0.05^{c}
**Protocatechuic acid**	0.98 ± 0.04^{a}	0.91 ± 0.03^{a}	0.82 ± 0.03^{b}	0.54 ± 0.02^{c}
**Vanillic acid**	2.05 ± 0.08^{a}	1.68 ± 0.06^{b}	1.35 ± 0.05^{c}	1.15 ± 0.04^{d}
Flavonoids and Other Compounds				
**Catechin**	19.80 ± 0.75^{a}	15.07 ± 0.57^{b}	7.25 ± 0.28^{c}	4.95 ± 0.19^{d}
**Epicatechin**	9.43 ± 0.36^{a}	7.16 ± 0.27^{b}	3.45 ± 0.13^{c}	2.12 ± 0.08^{d}
**Hydroxytyrosol**	0.89 ± 0.03^{a}	0.63 ± 0.02^{b}	0.42 ± 0.02^{c}	0.28 ± 0.01^{d}

Values are expressed as mean ± SD. Different superscript letters within the same row indicate statistically significant differences between samples ($p < 0.05$). ICR, Israeli Chardonnay; CCR, Chilean Chardonnay; ISB, Israeli Sauvignon Blanc; CSB, Chilean Sauvignon Blanc.

Table S2. Total phenolic content, tannin content, antioxidant capacity, and molar efficiency of Israeli and Chilean white wines used to construct the radar plot presented in Figure 1B.

Sample	TPC (mg GAE/L)	Tannins (mg/L)	CUPRAC (mmol TE/L)	DPPH (mmol TE/L)	Molar efficiency
**ICR**	440.5 ± 15.2^{a}	360.7 ± 12.8^{a}	2.91 ± 0.14^{a}	1.66 ± 0.07^{a}	6.61 ± 0.20^{a}
**CCR**	314.8 ± 11.5^{c}	268.7 ± 9.4^{c}	2.11 ± 0.10^{c}	1.22 ± 0.05^{c}	6.70 ± 0.30^{a}
**ISB**	360.8 ± 12.4^{b}	298.6 ± 10.2^{b}	2.34 ± 0.11^{b}	1.36 ± 0.05^{b}	6.48 ± 0.20^{b}
**CSB**	244.4 ± 9.8^{d}	197.8 ± 7.5^{d}	1.62 ± 0.08^{d}	0.98 ± 0.04^{d}	6.63 ± 0.30^{a}

Abbreviations: ICR: Israeli Chardonnay; CCR: Chilean Chardonnay; ISB: Israeli Sauvignon Blanc; CSB: Chilean Sauvignon Blanc; TPC: Total Phenolic Content; GAE: Gallic Acid Equivalents; CUPRAC: Cupric Ion Reducing Antioxidant Capacity; DPPH: 2,2-diphenyl-1-picrylhydrazyl; TE: Trolox Equivalents. Values are expressed as mean ±SD. Different superscript letters (a-d) within the same column indicate statistically significant differences ($p < 0.05$).

Table S3. Thermodynamic, fluorescence-quenching, and binding parameters of wine-derived polyphenols toward human serum albumin used to construct Figure 4.

Sample	Kb ($\times 10^4$ M^{-1})	ΔG (kJ/mol)	KSV proxy	Binding sites (n)	Binding Affinity Index (BAI)	Antioxidant Efficiency (AE)
**ICR**	8.44 ± 1.84a	−35.03 ± 0.31a	0.000558	0.26 ± 0.01a	11.89	0.00377
**CCR**	8.32 ± 1.75a	−34.82 ± 0.28a	0.000582	0.44 ± 0.02b	12.69	0.00388
**ISB**	4.05 ± 1.20c	−28.20 ± 0.19c	0.000438	0.57 ± 0.03c	10.04	0.00377
**CSB**	2.81 ± 1.05d	−25.70 ± 0.15d	0.000437	0.69 ± 0.04d	9.85	0.00401
**Ethanol**	0.85 ± 0.05e	−5.12 ± 0.20e	0.000085	n/a	1.15	0.00012

Values are expressed as mean ± SD where applicable. Different superscript letters within the same column indicate statistically significant differences between samples ($p < 0.05$). ICR, Israeli Chardonnay; CCR, Chilean Chardonnay; ISB, Israeli Sauvignon Blanc; CSB, Chilean Sauvignon Blanc; Kb, binding constant; ΔG, Gibbs free energy change; KSV, Stern–Volmer quenching constant proxy; n, apparent number of binding sites per protein molecule; BAI, Binding Affinity Index; AE, antioxidant efficiency. Ethanol was used as the negative control.

Table S4. Binding and thermodynamic parameters of selected wine-related phenolic standards toward human serum albumin used to construct the radar plot.

Standard compound	Kb ($\times 10^4$ M^{-1})	ΔG (kJ/mol)	Binding sites (n)	Binding affinity
**Epicatechin**	8.91 ± 0.30a	−5.420 ± 0.165a	0.88 ± 0.04	Very high
**Catechin**	8.12 ± 0.25a	−5.190 ± 0.150a	1.09 ± 0.05	Very high
**Caftaric acid**	7.54 ± 0.22b	−4.920 ± 0.140b	0.94 ± 0.06	High
**Caffeic acid**	7.22 ± 0.22b	−4.850 ± 0.140b	0.95 ± 0.06	High
**Gallic acid**	6.21 ± 0.18c	−4.120 ± 0.110c	1.10 ± 0.08	Moderate
**Protocatechuic acid**	5.88 ± 0.15c	−3.940 ± 0.105c	1.05 ± 0.07	Moderate
**p-Coumaric acid**	5.42 ± 0.14d	−3.710 ± 0.095d	1.02 ± 0.06	Moderate
**Vanillic acid**	4.91 ± 0.12d	−3.450 ± 0.090d	1.08 ± 0.09	Low
**Hydroxytyrosol**	4.15 ± 0.11e	−3.120 ± 0.085e	1.12 ± 0.10	Low

Values are expressed as mean ± SD (n = 25). Different superscript letters within the same column indicate statistically significant differences between compounds ($p < 0.05$). Kb, binding constant; ΔG, Gibbs free energy change; n, estimated number of binding sites per protein molecule.
